# Effects of Prehabilitation Concurrent Exercise on Functional Capacity in Colorectal Cancer Patients: A Systematic Review and Meta-Analysis

**DOI:** 10.3390/healthcare13101119

**Published:** 2025-05-12

**Authors:** Sergio Maroto-Izquierdo, Iker J. Bautista, Adriana Pérez-Guerrero, Paula Redondo-Delgado, Irati Jauregui-Fajardo, Vicente Simó, César Aldecoa

**Affiliations:** 1 i+HeALTH Strategic Research Group, Department of Health Sciences, European University Miguel de Cervantes, 47012 Valladolid, Spain; 2 Proporción A, Applied Sports Science Centre, 47015 Valladolid, Spain; 3 Institute of Sport and Allied Health, University of Chichester, Chichester PO19 6PE, UK; 4 Colorectal Surgery Unit, Hospital Universitario Rio Hortega, 47012 Valladolid, Spain; 5 Department of Anesthesiology, Facultad de Medicina de Valladolid, Hospital Universitario Rio Hortega, 47012 Valladolid, Spain

**Keywords:** pre-surgery, resistance exercise, aerobic exercise, coadjutant therapy, oncology patients

## Abstract

**Purpose**: Our aim was to examine the efficacy of concurrent exercise (i.e., aerobic and strength exercise) during prehabilitation programs on functional capacity in comparison with standard cancer care strategies in colorectal cancer (CRC) patients scheduled for surgery. **Methods**: A systematic review of randomized controlled trials was performed. A search of electronic databases [PubMed, Web of Science, and EBSCO Host] was conducted to identify all publications employing concurrent exercise in patients with CRC. Random-effects meta-analyses were used to calculate the standardized change in mean difference (SMD) and 95%CI between exercise intervention and control groups for the 6 min walking test (6MWT) distance covered before and after prehabilitation. **Results**: Six studies met the inclusion criteria (379 patients with CRC). Concurrent training during prehabilitation led to significant positive effects on the 6MWT (0.28 SMD [0.03–0.54], *p* = 0.037). Subgroup analyses showed a higher SMD (0.48 [0.00–0.98], *p* = 0.050) in younger (i.e., <70 years) CRC patients compared to their older counterparts (0.10 [0.08–0.11], *p* = 0.310). Meta-regression models between SMD of the 6MWT and body mass index, prehabilitation program duration, and baseline 6MWT distance covered did not show any significant relationship. **Conclusions**: This meta-analysis demonstrates the superiority of concurrent exercise prehabilitation in improving functional capacity related to cardiometabolic changes and lowering postoperative risk in patients with CRC.

## 1. Introduction

Colorectal cancer (CRC) is the third most common cancer in the world. More than 1.9 million new cases were diagnosed in 2022, approximately 10% of all annually diagnosed cancers, with an incidence rate of 67.4 per 100,000 persons, with a rising tendency [1]. The average age at CRC diagnosis is 66 years, and the incidence rate increases rapidly with age. In addition, the incidence rate is reported to be higher in men than in women (40–50% higher), especially in older people (55–74 years) [2]. However, despite causing more than 900,000 deaths worldwide in 2022 and being the third most common cause of cancer death [1], there has been a decrease in the mortality index and an increase in the survival rate over the last 20 years. This is attributed to advances in imaging techniques and diagnostic methods. In addition, improvements in treatment options, including enhanced surgical techniques, have played a significant role [2].

Despite these advancements, surgery is associated with a decline in functional capacity, particularly in older patients [3]. Postoperative complications are associated with increased in-hospital mortality and a higher probability to remain hospitalized [4]. Complications related to CRC surgery include adverse postoperative implications and a negative impact on daily quality of life [5]. Indeed, lower preoperative health and fitness levels are associated with surgical complications [6] and long-term function after major surgery in patients with CRC [3]. Thus, low levels of physical activity are associated with poor prognosis in patients with CRC [7]. Patients can even experience deterioration in their functional capacity while awaiting surgery [8,9]. Hence, it is important to propose healthcare strategies to fight surgery-induced adverse effects on functional capacity. Given the fact that the higher the number of risk factors (e.g., the type of surgery, baseline levels of physical activity, American Society of Anesthesiologists (ASA) grade, and age), the higher the risk of postoperative complications, preventive strategies are needed [10]. Prehabilitation offers a feasible and efficient strategy for improving postoperative outcomes related to modifiable risk factors [6].

Exercise interventions have been shown to be associated with a low risk of adverse events, leading to beneficial effects on a wide range of health-related outcomes in patients with CRC, including quality of life, fatigue, sleep, depression, aerobic fitness, functional strength, body fat, and decreased mortality [11]. Traditionally, aerobic low- to moderate-intensity activities (e.g., walking) have been proposed as a feasible and safe postoperative exercise intervention for CRC survivors, considering the general recommendation of 150 min/week [12,13,14,15,16]. Therefore, aerobic exercise is usually also prescribed to improve functional capacity and physical activity levels in patients before surgery [17,18] as a prehabilitation strategy.

The impact of prehabilitation interventions based on low- to moderate-intensity aerobic exercise in patients with CRC has been analyzed in numerous studies using the six-minute walk test (6MWT) [19]. This assessment allows for an understanding of the patients’ functional capacity [20,21,22,23,24,25,26], which is crucial in reducing disease recurrence and mortality [27,28]. Thus, to increase functional capacity, both aerobic and resistance exercises have been recommended for patients [29]. Indeed, the benefits of the 6MWT have been observed after 1–4 weeks of concurrent training during prehabilitation in cancer patients undergoing tumor resection [30]. Thus, recent CRC prehabilitation programs that include both exercise modalities have been shown to be the most efficient in generating gains in functional capacity before surgery [20,22,23,31] and have led to higher physical activity levels in patients with CRC [31]. This, in turn, was associated with the most successful postoperative recovery [32] and reduced lean tissue muscle mass loss induced by surgical stress [33].

Given the proven benefits of concurrent training in patients with CRC, prehabilitation programs including both aerobic and resistance exercise training may be associated with higher gains in functional capacity and cardiometabolic health-related factors prior to surgery than standard care. Therefore, the aim of this systematic review and meta-analysis was to analyze the effects of concurrent exercise programs during prehabilitation on functional capacity assessed using the 6MWT compared with traditional low- to moderate-intensity aerobic exercise traditionally prescribed along with standard cancer care strategies in patients with CRC scheduled for surgery.

## 2. Materials and Methods

A systematic review of the literature was performed using the guidelines in the Cochrane Handbook for Systematic Reviews of Interventions (version 6.0) following the checklist for the Preferred Reporting Items for Systematic reviews and Meta-Analyses 2020 (PRISMA) [34]. The PRISMA statement includes a 27-item checklist that details reporting recommendations for each item and is designed to be used as a basis for reporting systematic reviews of randomized trials (Supplementary File S1). The review protocol was registered in the PROSPERO database (registration number CRD42022307792).

### 2.1. Study Characteristics

A systematic, computerized search of the literature in PubMed, Web of Science (including Web of Science and MEDLINE results) and EBSCO Host (CINAHL with Full Text, eBook Collection (EBSCOhost), ERIC, Food Science Source, Library, Information Science & Technology Abstracts, MEDLINE Complete, PSICODOC, SPORTDiscus with Full Text) was conducted by an independent researcher with controlled vocabulary and keywords related to colorectal cancer prehabilitation and concurrent exercise. Our search time frame was restricted to 17 years (January 2009 to March 2025); 2009 was chosen because research on exercise-based prehabilitation programs for patients with CRC began that year [18]. A PICOC systematic search strategy was developed for PubMed using a word frequency analyzer tool (http://sr-accelerator.com/#/help/wordfreq (accessed on 12 March 2025)) to identify potentially relevant search terms. The Research Refiner tool (https://ielab-sysrev2.uqcloud.net/ (accessed on 12 March 2025)) was subsequently used to optimize the sensitivity and specificity of the search, and the Polyglot Search Translator Tool (https://sr-accelerator.com/#/polyglot (accessed on 12 March 2025)) was used to adapt the search to another database:

Population: Patients diagnosed with CRC.

Intervention: Concurrent exercise prehabilitation programs, which include both aerobic and resistance training.

Comparison: Standard cancer care strategies without structured exercise programs.

Outcome: Improvement in functional capacity assessed though the 6MWT.

Context: Preoperative phase in patients scheduled for colorectal cancer surgery.

### 2.2. Search Method of Studies

The search language was restricted to English, and a filter containing Medical Subject Headings (MeSH) terms was used. A more specific search included the terms “Prehabilitation”, “training”, “fitness”, “resistance”, “strength”, “weight”, “cancer”, “colon”, “rectal”, “colorectal” and “Oncology.” Thus, the following search string was: “Prehabilitation” [All Fields] AND (“training” [All Fields] OR “fitness” [All Fields] OR “resistance” [All Fields] OR “strength” [All Fields] OR “weight” [All Fields]) AND (“cancer” [All Fields] OR “colon” [All Fields] OR “rectal” [All Fields] OR “colorectal” [All Fields] OR “oncology” [All Fields]). The search strings used for each database are detailed in Supplementary File S2.

The reference lists of all selected publications were verified to retrieve relevant publications that were not identified by the computerized search. References to the selected publications included original articles, abstracts, and conference proceedings. To identify relevant articles, the titles and abstracts of all publications selected after the initial search were analyzed in search of prehabilitation strategies involving some form of exercise for CRC patients. Thus, in addition to the titles identified in the initial search, the titles and abstracts of all newly recognized publications that included strategies for prehabilitation with some form of exercise for CRC patients were examined in detail. Studies were excluded if their titles and abstracts did not include reference to the use of prehabilitation strategies with physical activity for colorectal cancer patients or if they did not meet other eligibility criteria, such as study design or population. Full-text papers were recovered if the abstract provided insufficient information to establish eligibility or if the article abstract had passed the first eligibility review.

### 2.3. Selection of Studies

All articles examining any exercise intervention as prehabilitation in colorectal cancer patients were eligible for full-text review. An article was eligible for inclusion if it met all of the following criteria:

1. The original article was a randomized controlled trial (RCT) or clinical controlled trial published in peer-reviewed journals.
2. The article reported patients with CRC of either sex who had completed a prehabilitation protocol during at least 2 weeks with a minimum training frequency of 2 days per week.
3. The manuscript included a prehabilitation intervention based on concurrent exercise and a control or exercise-based alternative intervention group, comparing functional capacity.
4. The prehabilitation program included strength and moderate- to high-intensity aerobic exercises.
5. The main outcome considered for analysis was 6MWT.

An article was excluded if the following applied:

6. Participants with any other cancer type, severe pathologies, or subjects with existing, or under treatment for, musculoskeletal injuries.
7. Did not have minimum requirements regarding the prehabilitation protocol (e.g., duration or frequency).
8. Reports focused on healthy subjects.
9. Not written in English.

The eligibility criteria were applied independently by two reviewers (CA and SMI) throughout the screening process, including review of titles, abstracts and full texts of articles. One researcher (IJB) reviewed the included articles and refined the eligibility criteria to narrow the scope of this review. Duplicate references were removed using an online deduplication tool for systematic reviews (https://sr-accelerator.com/#/libraries/dedupe (accessed on 12 March 2025)) and manually checked. Systematic review software (Rayyan, https://www.rayyan.ai; accessed on 12 March 2025) was used for screening, with blinding implemented to minimize bias. Disagreements were resolved through discussion or, if necessary, by a third reviewer (IJB).

### 2.4. Data Extraction and Management

Data extraction was performed independently and in duplicate by two authors (CA and SMI). The data were then merged by one author (CA), and any discrepancies in the extracted data were resolved through discussion or by a third reviewer (IJB), if required. Extracted data from each full-text article included the following: (1) study identification information; (2) study design; (3) sample size; (4) sex and ethnicity; (5) age, height, and body mass; (6) exercise program characteristics (e.g., program duration, weekly training frequency and training volume, exercises prescribed, exercise intensity, training load management, and supervision); (7) 6MWT distance covered at baseline and before surgery; (8) means and standard deviations for relevant outcome measures (i.e., pre- and post-test 6MWT performance); and (9) exact *p*-values, r-values, t-values, or confidence intervals for an association between two outcomes or a comparison between groups. When insufficient data were reported, authors were contacted via email. When data were not presented in tables or text, and when authors did not provide the requested data, they were extracted from figures using WebPlot Digitizer (https://automeris.io/WebPlotDigitizer, accessed on 12 March 2025) when possible.

### 2.5. Assessment of Risk of Bias

Methodological quality and risk of bias were independently assessed by two researchers (CA and SMI) using Cochrane Risk of Bias 2 (RoB2). In cases of disagreement between the scores, a third author made the final decision (IJB). The RoB2 assessment scale was structured into a fixed set of domains of bias, focusing on different aspects of the trial design, conduct, and reporting. Five domains were assessed: (D1) bias arising from the randomization process, (D2) bias due to deviations from intended interventions, (D3) bias due to missing outcome data, (D4) bias in the measurement of the outcome, and (D5) bias in selection of the reported results. These categories were classified as “high risk of bias”, “low risk of bias”, or “some concerns”. In addition to evaluating individual study quality using the Cochrane Risk of Bias 2 (RoB2) tool, we also employed the Grading of Recommendations Assessment, Development, and Evaluation (GRADE) system. The GRADE system allowed us to assess the overall certainty of evidence across the included studies by examining five key dimensions: RoB (as assessed by RoB2), inconsistency, indirectness, imprecision, and publication bias. The integration of GRADE provided a comprehensive appraisal of how confidently our meta-analysis findings represent true effects, thereby guiding informed clinical recommendations. Briefly, the overall quality was rated as high and downgraded to moderate, low, or very low for each of the following limitations: For imprecision, the level of evidence was downgraded to one to determine whether the conclusion about the effect magnitude would be altered based on the lower or upper boundary of the confidence interval. For example, if the mean effect was small but the 95% confidence interval crossed the threshold for a trivial effect (i.e., g < 0.2), the precision was insufficient to support a strong recommendation, and the confidence interval did not exclude the possibility that the effect was trivial. Similarly, if the confidence interval crossed the threshold for a large effect, whereas the mean effect was moderate, the conclusion was considered imprecise, and, as such, the level of evidence was downgraded to one level. For inconsistency, the level of evidence was downgraded to one level if high statistical heterogeneity was observed and if more than 50% of the studies had >1 risk of bias item assessed as high risk. Finally, no indirectness rating was applied because all the studies had a similar sample (i.e., CRC patients undergoing intervention in the following 2–4 weeks), all of them used the same tool to assess functional capacity, and interventions were similar, including both resistance and aerobic exercise forms. Additionally, indirect measurements were not performed.

### 2.6. Statistical Analysis

A random-effects meta-analysis was conducted to compute the standardized mean difference (SMD) and its 95% confidence intervals (CI) between intervention and control groups. SMD was considered trivial (<0.20), small (0.20–0.59), moderate (0.60–1.19), large (1.20–1.99), or very large (>2.00) [35]. Variance estimations were calculated using the Hartung–Knapp/Sidik–Jakman adjustment, and heterogeneity was assessed using I^2^ and Tau-square (τ^2^) tests. Prediction intervals were included to evaluate the clinical implications of heterogeneity and the probability of true-positive effects [36]. Publication bias was assessed using contour-enhanced funnel plots and a p-curve analysis. Statistical analyses were performed using R software (version 4.1.9), and risk of bias figures were created using Robvis software (https://www.riskofbias.info/welcome/robvis-visualization-tool, accessed on 12 March 2025).

## 3. Results

### 3.1. Selection of Studies

**Figure 1:**
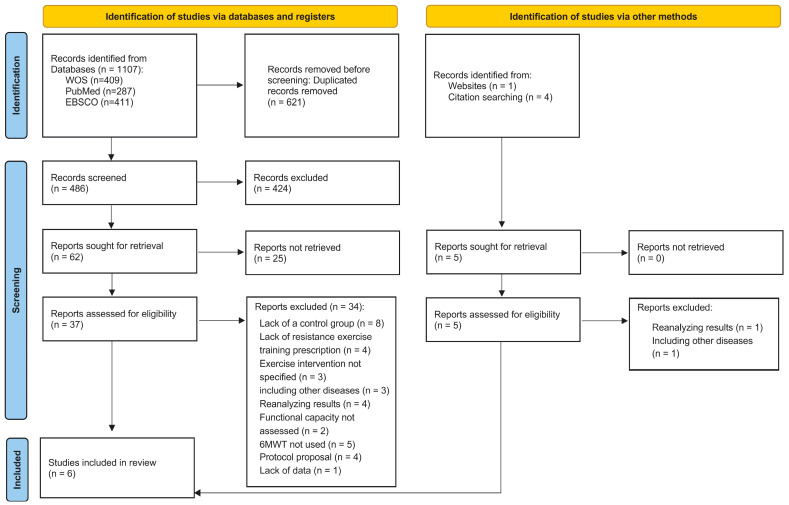
Flow chart of the different phases of the search and selection of studies included in this review.

Figure 1 shows a flow chart of the different phases of the search and selection of studies included in this review. The initial search of electronic databases identified 1640 titles, of which 959 were rejected for duplication issues. Five titles/articles [9,20,25,31,37] were identified through a manual search. Thus, 681 titles were identified, but 578 were rejected after reading the titles because they did not include reference to the use of prehabilitation strategies with physical activity for patients with CRC or they did not meet other eligibility criteria, such as study design or population. From a total of 108 abstracts that were screened, 73 were excluded because they did not meet inclusion criteria: 41 studies were reviews, 7 studies analyzed prehabilitation on other types of cancer, 3 studies were excluded due to applying a nutritional intervention, 2 studies were nursing standards, 2 studies were not available, 2 studies did not include a control group, 7 studies were not written in English, and 9 studies were not related to CRC prehabilitation. Thus, forty-two full texts were reviewed, but only six studies satisfied the inclusion criteria for this review [20,21,23,24,25,37]. The main reasons for exclusion included lack of a comparison group (*n* = 8), intervention did not include resistance training (*n* = 4) or did not specify the type of exercise intervention (*n* = 3), included diseases other than CRC and did not differentiate results (*n* = 3), reanalyzed results from previous controlled trials (*n* = 4), did not measure functional capacity (*n* = 2), did not assess functional capacity through the 6MWT (*n* = 5), an intervention protocol proposal (*n* = 4), or lack of data (*n* = 1). The complete list of excluded references and rationale for their exclusion can be found in Supplementary File S3. The RoB2 scores of the included studies are presented in Supplementary File S4. Visual inspection of the contour-enhanced funnel plots and Egger’s test indicated no asymmetry in the 6MWT.

### 3.2. Characteristics of Included Studies

The main characteristics of the studies included in this review, including the participants, interventions, and results, are presented in Table 1. The six included studies involved a total of 379 participants, with 192 (50.7%, 110 men and 82 women) in the prehabilitation group and 187 (49.3%, 106 men and 81 women) in the control group. Participants had a mean age of 70.4 ± 6.2 years, and the majority were overweight (mean BMI: 27.5 ± 1.4). All studies assessed functional capacity using the 6MWT, with baseline distances ranging from 358.2 ± 21.7 m in the control group to 404.1 ± 41.8 m in the prehabilitation group. All studies measured the functional walking capacity using the 6MWT. In addition, the stair climb test, the five times sit-to-stand test [20], the chair stands in 30 s test [24], the time up and go test, handgrip strength [20], habitual and maximal gait speed, and inspiratory muscle strength [24] were also measured. However, these results were not analyzed in the present study.

Prehabilitation interventions ranged from 2 to 6 weeks, with a frequency ranging from two to four sessions per week, with three sessions per week the most common paradigm. Every study included both resistance and aerobic training, but not all provided details about the intervention. Aerobic and resistance training was performed within the same session in five studies [20,21,23,24,37], and the duration ranged from 50 [23] to 60 min [20,21,24]. Only one study did not specify the prescription of aerobic and resistance training within the same session [25]. Training sessions usually took place at patients’ residences [21,23,24,25,37], including hospital sessions once a week [21,37]. Only one study performed a supervised exercise program conducted by researchers in the laboratory [20]. Training sessions were totally unsupervised in two studies [23,25], unsupervised but included a supervised session once a week in one study [21,37] or totally supervised in two studies [20,24]. Most studies performed moderate-intensity aerobic exercise for 20–30 min [20,21,23,25,37], which included exercises such as walking/jogging [20,21,23,25,37], cycling [21,23], swimming [23], using a recumbent stepper [37], or using an aerobic exercise device [25], except for one that used high-intensity interval aerobic training with exercises such as brisk walking [24]. Resistance training ranged from to two to four sets and 8 to 15 reps [20,21,23,24]. Exercises targeted major muscle groups [21,23], functional strength exercises [24], weightbearing and elastic band exercises [25,37] and a variety of exercises using body weight, resistance bands, kettlebells, dumbbells, and balls [20].

### 3.3. Main Effects of Concurrent Exercise

**Figure 2:**
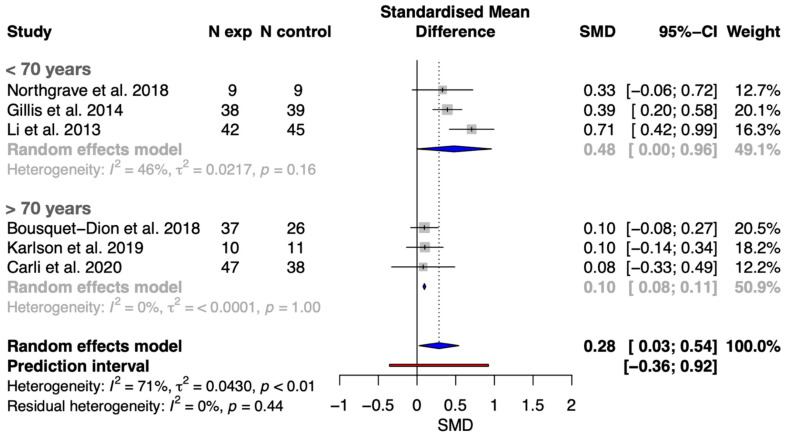
Forest plot with meta-analysis of SMCD showing comparison of concurrent exercise prehabilitation versus standard cancer care on functional capacity (assessed through the 6MWT) in younger (<70 years) and older (>70 years) patients with CRC [20,21,23,24,25,37].

Regarding the meta-analysis on the effects of concurrent exercise compared to traditional care strategies on distance covered in the 6MWT, the results showed statistically significant differences (t-value = 2.83, *p* = 0.037) by 0.28 SMD [0.03, 0.54] in favor of the intervention group (i.e., implementation of aerobic and strength exercise during prehabilitation). The prediction interval and heterogeneity are illustrated in Figure 2, and the GRADE quality evidence is provided in Supplementary File S5. The prediction interval revealed that concurrent exercise during prehabilitation interventions has a probability of a true-positive effect of 0.85 in a future setting. The counter-enhanced funnel plot and p-curve analysis showed no evidence of publication bias (Supplementary Files S6 and S7, respectively). Visual inspection of the counter-enhanced funnel plots for distance in the 6MWT showed no large asymmetries (see Supplementary File S6). In addition, a p-curve analysis was used as an alternative method to assess publication bias. Supplementary File S7 shows the p-curve (in blue), Right-Skewness test, and Flatness test for the 6MWT, when including all distances.

### 3.4. Meta-Regression Models

**Figure 3:**
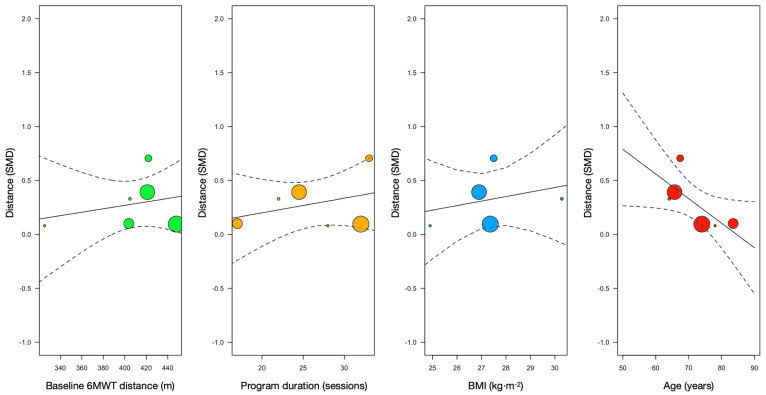
Meta-regression models of distance covered at baseline, program duration (i.e., number of sessions before surgery), body mass index (BMI), and age with SMCD of 6MWT distance (vertical axis).

Meta-regression models showed that body mass index, age, intervention duration (sessions), and 6MWT distance covered at baseline were not significantly associated with improvements in the 6MWT distance effect size (see Table 2). However, there was a positive trend towards age in the effect size of distance (Figure 3).

## 4. Discussion

This systematic review and meta-analysis demonstrated that concurrent exercise prehabilitation (combining aerobic and resistance training) leads to significant improvements in functional capacity, as measured by the 6MWT, compared to standard care in patients with CRC scheduled for surgery. Our meta-analysis, which included six studies and 379 patients, found a small but significant effect size (SMD = 0.28 [0.03 to 0.54]) in favor of concurrent exercise interventions. Additionally, meta-regression analyses explored the influence of age, BMI, baseline walking ability, and program duration on these outcomes. In addition, subgroup analysis and meta-regression models showed that age, and likely age-related variables, seems to be a key factor in prehabilitation effectiveness on functional capacity, since younger (i.e., <70 years) patients with CRC showed larger effects compared to their older counterparts (SMD 0.48 [0.00 to 0.98] vs. 0.10 [0.08 to 0.11]).

Surgical complications in CRC are closely linked to preoperative physical status [6,38,39], making prehabilitation strategies, especially those including exercise [21,22,23,25,37,40,41], essential to improve patients’ functional capacity and reduce perioperative risk [11]. The 6 min walk test (6MWT), used in all studies included in this review, is a practical and validated tool for assessing functional capacity in cancer patients [42,43] and correlates with postoperative recovery [31,44,45,46]. Although improvements in 6MWT performance do not always guarantee better clinical outcomes, increasing walking capacity is a modifiable risk factor that may positively influence recovery. Future research should clarify the relationship between functional gains and clinically relevant outcomes and explore whether longer prehabilitation windows, such as in patients receiving neoadjuvant treatment, can further enhance both functional and clinical results.

This systematic review and meta-analysis found that exercise-based prehabilitation significantly improved functional capacity in CRC patients younger than 70 years (SMD 0.48 [0.00 to 0.98]), while the effect in those older than 70 years was trivial (SMD 0.10 [0.08 to 0.11]). Although no previous studies have specifically analyzed the association between age and training-induced effects in this context, our results suggest that younger patients benefit more, possibly due to fewer age-related comorbidities, lower prevalence of frailty and sarcopenia, and greater adaptive capacity. Frailty and sarcopenia, common in older patients [46], are linked to higher postoperative risk and may limit the effectiveness of prehabilitation [47]. Notably, poorer baseline physical status did not predict greater improvements in older patients, in contrast to findings in younger cohorts [23,25]. Therefore, tailoring exercise programs to individual characteristics, such as age, baseline fitness, and comorbidities, may enhance prehabilitation outcomes. For instance, older patients might benefit from lower-intensity, longer-duration protocols, while younger or fitter patients may respond better to higher-intensity programs. Although a previous review found the greatest mortality reduction in CRC survivors with BMI < 25 [11], our analysis did not show a significant association between BMI and functional improvements, possibly due to limited reporting on body composition (e.g., lean mass, fat percentage) in the included studies [48,49]. Further research should focus on optimizing interventions for older patients and incorporating more precise body composition measurements.

Our analysis showed no significant correlation between the duration of the prehabilitation program and the gains achieved. However, this could be due to the short and similar time periods between the interventions (2–4 weeks). A previous meta-analysis on exercise interventions in patients with CRC found no significant effects on cardiovascular fitness after interventions that lasted <12 weeks but did find significant effects for interventions that lasted 12 weeks or longer, leading to the assumption that the longer the prehabilitation interventions, the greater the changes [50]. However, the duration of prehabilitation is usually determined by the time to surgery, which limits prehabilitation to a very short time frame but is sufficient to improve preoperative walking ability [31]. Nonetheless, a longer delay in treatment has been reported to not result in lower overall or cancer-free survival in patients with primary CRC undergoing curative surgical treatment, again supporting the idea of adjusting waiting times to implement effective preoperative programs [51]. However, the lack of a positive correlation between a longer prehabilitation program and greater effects on functional capacity found in this study suggests that other variables of the training program, such as intensity, exercise selection or weekly training frequency, need to be modified. Resistance training is a unique form of training that can improve not only functional capacity but also muscle mass, fat percentage and fat distribution in cancer patients undergoing neoadjuvant and adjuvant therapy [52]. Therefore, considering that concurrent training is recommended during cancer treatment [29], its inclusion in combination with aerobic exercise in the CRC prehabilitation programs appears to be essential [20,22,23,25,53,54,55,56,57,58].

Consequently, the most important variable to influence when prescribing exercise for patients with cancer is intensity. As the included studies did not specify exercise intensity, it was not possible to compare the changes achieved in the 6MWT following an exercise-based prehabilitation intervention and exercise intensity. However, previous research has shown that moderate- to vigorous-intensity training interventions can lead to an improvement in fitness level at VO2peak [59], muscle strength and endurance [59], functional capacity (including 6MWT) [60], as well as measures of immune and cognitive function, depression, and anxiety. Further research is needed, particularly in the area of resistance training, to determine the percentage of 1-RM or the bar velocity (i.e., specific intensity), number of repetitions, total number of exercises or optimal weekly training volume for a precise exercise programs in both the prehabilitation and coadjutant treatment of cancer patients. However, increased exercise intensity requires the supervision of qualified healthcare providers. Supervised training produced better results than unsupervised programs and led to a significant reduction in the prevalence of adverse events [61].

The results of this systematic review and meta-analysis must be considered with some limitations. One of the main limitations is the small number of studies (k = 6) that met the eligibility criteria and could subsequently be included in the meta-regression models (a smaller number than recommended in the Cochrane guidelines [62] for conducting meta-regression models). In addition, there were few studies in the overall scientific literature that analyzed the effects of concurrent training during prehabilitation in patients with CRC. Furthermore, interventions were heterogeneous, as some were described as multimodal prehabilitation, while others included only an exercise component. Differences in exercise prescription, such as intensity, volume, frequency, and progression, may have contributed to the variability in outcomes. In addition, patient characteristics, such as age, baseline fitness level, comorbidities and BMI, likely influenced the observed results, as these factors may influence the individual response to exercise interventions. The quality of the studies also varied, with some studies having a high risk of bias, which may have affected the reliability of the results. Despite all the included studies in this review, the prescribed aerobic and resistance training, adherence and compliance or standard treatment methods varied between studies. In addition, none of the studies reported in detail on the exercise intervention. This suggests that the results may have been confounded by many other factors, such as exercise intensity or exercise management strategies. Future research should further investigate the effects of different exercise intensities during prehabilitation in colorectal cancer patients. Although our meta-regression analyses did not find significant associations between age, BMI, baseline walking capacity, or program duration and the effect size, these factors may still influence individual responses to prehabilitation. Importantly, the RoB assessment revealed concerns related to deviations from intended interventions, particularly regarding adherence and implementation fidelity, which could affect the reliability of the results. Therefore, our findings should be interpreted with caution. Future research should prioritize rigorous reporting of intervention adherence and implementation, as well as the use of intention-to-treat analyses, to strengthen the evidence base. Overall, our results support the integration of concurrent exercise prehabilitation into standard care for CRC patients, but further high-quality studies are needed to confirm these benefits and to optimize program design for different patient subgroups.

## 5. Conclusions

In conclusion, although moderate-intensity aerobic activities are the most popular mode of exercise during prehabilitation in cancer patients, the results of this systematic review and meta-analysis indicate that their combination with resistance exercise (i.e., concurrent exercise) is associated with greater improvements in functional capacity, assessed through the 6MWT, in patients with CRC. Therefore, implementing concurrent exercise three to four times a week for 2–4 weeks before surgery is recommended to increase walking capacity in patients with CRC, which may contribute to improved preoperative fitness. In addition, subgroup analysis demonstrated that younger patients (i.e., <70 years) showed greater changes in walking ability than their older counterparts. Meta-regression models showed that training-induced effects were not dependent on baseline 6MWT distance covered, intervention duration, or BMI. Thus, to fully understand the effects of concurrent exercise during the preoperative period, future research should involve individualized programs based on patients’ biometric and clinical characteristics.

## Figures and Tables

**Table 1:** Main characteristics of the studies included.

Study	Participants	Functional Outcomes	Program Supervision	Exercise Intervention	Other Interventions	Results
Bousquet-Dion et al. [21] Canada	63 Prehab (*n* = 37), Rehab (*n* = 26)	6MWT	Non-supervised home-based program +a weekly session supervised at hospital	Average duration: 32 days, 3–4 days/week. Session: 30 min AE (including 5 min WU) + 25 min RT + 5 min stretching. AE intensity: 60–70% HRmax. AE activities: walking, cycling or jogging. RT: 8 exercises targeting major muscle groups. RT volume: 2 sets × 8–15 reps. RT was progressed (i.e., increasing intensity) when patients were able to complete the program with perceived mild exertion (12 RPE points or less on the 20-point Borg scale).	Nutritional intervention & anxiety reduction strategies	Both groups improved 6MWT over the preoperative period (Prehab: 4.9%, Rehab: 2.2%). However, Prehab group showed higher changes. Including a weekly supervised session did not provide higher functional enhancements compared to home-based multimodal prehabilitation programs.
Carli et al. [37] Canada	110 Prehab (*n* = 55), Control (*n* = 55)	6MWT	Non-supervised home-based program +a weekly session supervised at hospital.	Duration: 28 days. 4 days/week. Supervised session: 30 min moderate AE (including a 5 min WU) on a recumbent stepper + 25 min RT using elastic bands + 5 min of stretching. Home based program: walk daily for a total of 30 min at moderate intensity + RT (elastic band routine 3 times per week)	Nutritional & Psychological strategies	Both groups improved walking capacity over the preoperative period, however greater improvements were found in Prehab group (Prehab:6.4%; Control: 3.9%)
Gillis et al. [23] France	77 Prehab (*n* = 38), Rehab (*n* = 39)	6MWT	Non-supervised home-based program	Average duration: 24.5 days, 3 days/week, Session: 5 min WU + 20 min AE + 20 min RT + 5 min CD. AE intensity: prescribed based on the rate of perceived exertion (Borg scale) from the 6MWT, starting at 40% HRR. AE activities: Walking, jogging, swimming, or cycling. RT: 8 exercises targeting major muscle groups, 8–12 RM. Exercise intensity progressions occurred when participants reported mild exertion (Borg 12) during AE and/or when participants completed 15 repetitions of a given RT exercise.	Nutritional intervention & anxiety reduction strategies	The 6MWT distance was significantly improved in patients with CRC waiting for surgery (6.0%); while those in Rehab group declined their functional walking capacity (−3.9%) during the prehabilitation period.
Karlsson et al. [24] Sweden	21 Prehab (*n* = 10), Control (*n* = 11)	6MWT; habitual and maximal gait speed; lower extremity strength (chair stands in 30 s); and Inspiratory muscle strength.	Supervised home-based program	Average duration: 17 days, 2–3 days/week, Session: 60 min. Block I: Inspiratory muscle training, Block II: RT, Block III: AE. AE: Bouts of stair climbing, Nordic walking outdoors, and interval walking indoors and/or outdoors. Intervals (RPE of 7–8 on Borg CR-10). The duration of AE sessions, number and length of AE intervals were progressively increased. RT volume: 3 sets × 10 reps. RT intensity: RPE of 7–8 on Borg CR-10. RT exercises: functional exercises (chair stands and step-up with weight belts). RT progression: the chair-stand test was performed before each session to establish load and volume, which were increased whether RPE was lower than 7 on Borg CR-10. During unsupervised days, participants were instructed to follow the general recommendation of 150 min/week of moderate intensity AE, combined with unloaded functional RT exercises 2–3 times/week.	No	Trimodal program comprising homebased moderate AE and RT improved their functional walking capacity. (Prehab: 1.9%, Control: 1.0%). Prehab participants improved also lower limb strength (Prehab: 34.3%; Control: 12.2%); gait speed (Prehab: 13.7%; Control: 5.6%); and inspiratory muscle strength (Prehab: 24.7%; Control: 1.5%). However, maximal gait speed did not seem to change after intervention (prehab: −2.0%; control: 5.8%)
Li et al. [25] Canada	87 Prehab (*n* = 42), Control (*n* = 45)	6MWT	Non-supervised home-based program	Average duration: 33 days AE: 3 days/week, 30 min at 50% HRmax. AE activities: walking or using an aerobic machine. RT: 3 days/week, calisthenics and elastic band movements performed to volitional fatigue.	Nutritional intervention & anxiety reduction strategies	Short period of concurrent exercise prehabilitation program supposed improvements on functional walking capacity only in Prehab group (9.9%).
Northgraves et al. [20] United Kingndom	21 Prehab (*n* = 10), Control (*n* = 11)	6MWT; Time Up and Go test; Five Times Sit To Stand test; Stair Climb Test, and handgrip strength.	Supervised by researchers at laboratory.	Average duration: 22 days, 3 days/week. Session: 60 min. WU (5 min on cycle ergometry at 40–50% HRR) + RT circuit 2 + AE + RT circuit 1 + CD (5-min walking and stretching). AE: Up to 25 min of cyclergometry at 40–60% HRR and/or RPE of 11–13 on the Borg scale. As tolerated, duration of cycling was increased by 2–5 min per session up to a maximum duration of 25 min RT volume: 3–4 sets for both circuit 1 and 2. RT exercises: Circuit 1: Split squat, rear foot elevated squat, bilateral and unilateral gluteal bridge, cook hip lift, shoulders elevated bilateral and unilateral gluteal bridge, kettlebell swings, dumbbell push press, high kneeling band anti-rotation, band resisted side shuffles, suitcase carry, and slam ball throws. Circuit 2: Sit to stand, band resisted sit to stand, side lying leg hip abduction, X-band walks, foot raised thoracic extension, shoulder band pull apart, band resisted external rotation, and band seated row. RT Progressions: each 2–3 sessions based on participant’s exercise technique and participant-reported difficulty.	No	Both groups improved walking capacity over the preoperative period. However, Prehab group showed higher improvements (Prehab: 17.3%; Control: 1.9%). Concurrent exercise did not improve any of the other measures except for handgrip strength (5.1%).

Abbreviatures: 6MWT, the six-minute walking test; AE, aerobic exercise; Control: Control group; CD, cool-down; HRmax, Maximum heart rate; HRR; heart rate reserve; Prehab: prehabilitation group; RPE, rate of perceived exertion; RT, resistance training; WU, warm-up.

**Table 2:** Meta-regression models between SMD of 6MWT distance covered and body mass index (BMI), age, prehabilitation program duration, and baseline 6MWT distance covered in all studies evaluated (k = 6).

Meta-Regression Models	k	Coefficients	SE	CI_95%_	*p*-Value	R^2^ (%)
SMD of 6MWT and BMI	6	Int: −0.82	2.24	−7.96 to 6.32	0.738	0%
		Slope: 0.04	0.08	−0.29 to 0.51	0.644	
SMD of 6MWT and age	6	Int: 1.93	0.81	−0.33 to 4.19	0.076	54%
		Slope: −0.02	0.01	−0.05 to 0.01	0.111	
SMD of 6MWT and program duration	6	Int: −0.07	0.49	−1.43 to 1.29	0.891	0%
		Slope: 0.01	0.02	−0.03 to 0.06	0.499	
SMD of 6MWT and baseline 6MWT	6	Int: −0.38	1.26	−3.86 to 3.13	0.785	0%
		Slope: 0.002	0.003	−0.007 to 0.01	0.631	

Abbreviations: 6MWT, 6 min walking test; SMD, standardized change of mean difference.

## Data Availability

Data will be made available upon reasonable request.

## Supplementary Materials

The following supporting information can be downloaded at: https://www.mdpi.com/article/10.3390/healthcare13101119/s1, Supplementary File S1: PRISMA Checklist; Supplementary File S2: Detailed Search Strategy for Each Database; Supplementary File S3: Reasons for Excluding Papers and References; Supplementary File S4: Risk of Bias Assessment for Each Study and Total Scores; Supplementary File S5: Summary of Meta-Analysis Findings and GRADE Quality Evidence Synthesis; Supplementary File S6: Visual Inspection of Counter-Enhanced Funnel Plots for Distance in the 6MWT; Supplementary File S7: P-Curve Analysis (in Blue), Right-Skewness Test, and Flatness Test for the 6MWT; Supplementary File S8: Results for Right-Skewness Test and Flatness Test for 6MWT Distance. Refs. [63,64,65,66,67,68,69,70,71,72,73,74,75,76] are cited in the Supplementary Materials.
