# Supplementary material for: Effects of Prehabilitation Concurrent Exercise on Functional Capacity in Colorectal Cancer Patients: A Systematic Review and Meta-Analysis
Source: Healthcare (Basel). 2025 May 12;13(10):1119. doi: 10.3390/healthcare13101119 (PMC12110785; doi:10.3390/healthcare13101119)
Supplement: Supplementary file 1 [file healthcare-13-01119-s001.zip › Supplementary File 1.pdf]

The main reasons for exclusion were:

1. Lack of a comparison group (n=8) (1–8),
2. The intervention did not include resistance training (n=4) (9–12)
3. The study did not specify the type of exercise intervention (n=1) (13),
4. The study included diseases other than CRC and did not differentiate results (n=3) (14–16),
5. Reanalyzed results from previous controlled trials (n=3) (17–19),
6. Did not measure functional capacity (n=2) (20,21),
7. Did not assess functional capacity through the 6MWT (n=4) (22–25),
8. The study was an intervention protocol proposal (n=3) (26–28)
9. There was a lack of data (n=1) (29).

REFERENCES:

1. Alejo L, Pagola-Aldazabal I, Fiuza-Luces C, Huerga D, De Torres M, Verdugo A, et al. Exercise prehabilitation program for patients under neoadjuvant treatment for rectal cancer: A pilot study. J Cancer Res Ther. 2019;15(1):20-5.

2. Bruns ERJ, Argillander TE, Schuijt HJ, Van Duijvendijk P, Van Der Zaag ES, Wassenaar EB, et al. Fit4SurgeryTV At-home Prehabilitation for Frail Older Patients Planned for Colorectal Cancer Surgery: A Pilot Study. Am J Phys Med Rehabil. 2019;98(5):399-406.

3. Heldens AFJM, Bongers BC, de Vos-Geelen J, van Meeteren NLU, Lenssen AF. Feasibility and preliminary effectiveness of a physical exercise training program during neoadjuvant chemoradiotherapy in individual patients with rectal cancer prior to major elective surgery. Eur J Surg Oncol. 2016;42(9):1322-30.

4. Loughney L, Cahill R, O'Malley K, McCaffrey N, Furlong B. Compliance, adherence and effectiveness of a community-based pre-operative exercise programme: a pilot study. Perioper Med. 2019;8(1):1-12.

5. Minnella EM, Ferreira V, Awasthi R, Charlebois P, Stein B, Liberman AS, et al. Effect of two different pre-operative exercise training regimens before colorectal

surgery on functional capacity: A randomised controlled trial. Eur J Anaesthesiol. 2020;37(11):969-78.

6. Singh F, Newton RU, Baker MK, Spry NA, Taaffe DR, Galvão DA. Feasibility and Efficacy of Presurgical Exercise in Survivors of Rectal Cancer Scheduled to Receive Curative Resection. Clin Colorectal Cancer. 2017;16(4):358-65.

7. Singh F, Galvão DA, Newton RU, Spry NA, Baker MK, Taaffe DR. Feasibility and Preliminary Efficacy of a 10-Week Resistance and Aerobic Exercise Intervention During Neoadjuvant Chemoradiation Treatment in Rectal Cancer Patients. Integr Cancer Ther. 2018;17(3):952-9.

8. Suen M, Liew A, Turner JD, Khatri S, Lin Y, Raso KL, et al. Short-term multimodal prehabilitation improves functional capacity for colorectal cancer patients prior to surgery. Asia Pac J Clin Oncol [Internet]. abril de 2022 [citado 5 de abril de 2023];18(2). Disponible en: https://onlinelibrary.wiley.com/doi/10.1111/ajco.13564

9. Boereboom CL, Blackwell JEM, Williams JP, Phillips BE, Lund JN. Short-term pre-operative high-intensity interval training does not improve fitness of colorectal cancer patients. Scand J Med Sci Sports. 2019;29(9):1383-91.

10. West MA, Loughney L, Lythgoe D, Barben CP, Sripadam R, Kemp GJ, et al. Effect of prehabilitation on objectively measured physical fitness after neoadjuvant treatment in preoperative rectal cancer patients: A blinded interventional pilot study. Br J Anaesth. 2015;114(2):244-51.

11. Kim DJ, Mayo NE, Carli F, Montgomery DL, Zavorsky GS. Responsive measures to prehabilitation in patients undergoing bowel resection surgery. Tohoku J Exp Med. 2009;217(2):109-15.

12. Moug SJ, Mutrie N, Barry SJE, Mackay G, Steele RJC, Boachie C, et al. Prehabilitation is feasible in patients with rectal cancer undergoing neoadjuvant chemoradiotherapy and may minimize physical deterioration: results from the REx trial. Colorectal Dis. 2019;21(5):548-62.

13. Macleod M, Steele RJC, O'Carroll RE, Wells M, Campbell A, Sugden JA, et al. Feasibility study to assess the delivery of a lifestyle intervention (TreatWELL) for

patients with colorectal cancer undergoing potentially curative treatment. BMJ Open. 2018;8(6):1-11.

14. Valkenet K, Trappenburg JCA, Schippers CC, Wanders L, Lemmens L, Backx FJG, et al. Feasibility of Exercise Training in Cancer Patients Scheduled for Elective Gastrointestinal Surgery. Dig Surg. 2016;33(5):439-47.

15. Huang GH, Ismail H, Murnane A, Kim P, Riedel B. Structured exercise program prior to major cancer surgery improves cardiopulmonary fitness: a retrospective cohort study. Support Care Cancer. 2016;24(5):2277-85.

16. Carli F, Charlebois P, Stein B, Feldman L, Zavorsky G, Kim DJ, et al. Randomized clinical trial of prehabilitation in colorectal surgery. Br J Surg. 2010;97(8):1187-97.

17. Chabot K, Gillis C, Minnella EM, Ferreira V, Awasthi R, Baldini G, et al. Functional capacity of prediabetic patients: effect of multimodal prehabilitation in patients undergoing colorectal cancer resection. Acta Oncol. 3 de agosto de 2021;60(8):1025-31.

18. Chen BP, Awasthi R, Sweet SN, Minnella EM, Bergdahl A, Santa Mina D, et al. Four-week prehabilitation program is sufficient to modify exercise behaviors and improve preoperative functional walking capacity in patients with colorectal cancer. Support Care Cancer. 2016;25(1):33-40.

19. Minnella EM, Awasthi R, Gillis C, Fiore JF, Liberman AS, Charlebois P, et al. Patients with poor baseline walking capacity are most likely to improve their functional status with multimodal prehabilitation. Surg U S. 2016;160(4):1070-9.

20. Brunet J, Burke S, Grocott MPW, West MA, Jack S. The effects of exercise on pain, fatigue, insomnia, and health perceptions in patients with operable advanced stage rectal cancer prior to surgery: A pilot trial. BMC Cancer. 2017;17(1):1.

21. Loughney L, West MA, Dimitrov BD, Kemp GJ, Grocott MPW, Jack S. Physical activity levels in locally advanced rectal cancer patients following neoadjuvant chemoradiotherapy and an exercise training programme before surgery: a pilot study. Perioper Med. 2017;6(1):1-8.

22. Berkel AEM, Bongers BC, Kotte H, Weltevreden P, de Jongh FHC, Eijsvogel MMM, et al. Effects of Community-based Exercise Prehabilitation for Patients Scheduled for Colorectal Surgery With High Risk for Postoperative Complications. Ann Surg. 2021;Publish Ahead of Print(January).

23. Bojesen RD, Jørgensen LB, Grube C, Skou ST, Johansen C, Dalton SO, et al. Fit for Surgery—feasibility of short-course multimodal individualized prehabilitation in high-risk frail colon cancer patients prior to surgery. Pilot Feasibility Stud. 21 de enero de 2022;8(1):11.

24. Heil TC, Driessen EJM, Argillander TE, Melis RJF, Maas HAAM, Olde Rikkert MGM, et al. Implementation of prehabilitation in colorectal cancer surgery: qualitative research on how to strengthen facilitators and overcome barriers. Support Care Cancer. septiembre de 2022;30(9):7373-86.

25. Heil TC, Verdaasdonk EGG, Maas HAAM, van Munster BC, Rikkert MGMO, de Wilt JHW, et al. Improved Postoperative Outcomes after Prehabilitation for Colorectal Cancer Surgery in Older Patients: An Emulated Target Trial. Ann Surg Oncol. enero de 2023;30(1):244-54.

26. Amaro-Gahete FJ, Jurado J, Cisneros A, Corres P, Marmol-Perez A, Osuna-Prieto FJ, et al. Multidisciplinary Prehabilitation and Postoperative Rehabilitation for Avoiding Complications in Patients Undergoing Resection of Colon Cancer: Rationale, Design, and Methodology of the ONCOFIT Study. Nutrients. 3 de noviembre de 2022;14(21):4647.

27. Andersson M, Egenvall M, Danielsson J, Thorell A, Sturesson C, Soop M, et al. CANOPTIPHYS study protocol: Optimising PHYSical function before CANcer surgery: effects of pre-operative optimisation on complications and physical function after gastrointestinal cancer surgery in older people at risk—a multicentre, randomised, parallel-group study. Trials. 19 de enero de 2023;24(1):41.

28. Van Rooijen S, Carli F, Dalton S, Thomas G, Bojesen R, Le Guen M, et al. Multimodal prehabilitation in colorectal cancer patients to improve functional capacity and reduce postoperative complications: The first international randomized controlled trial for multimodal prehabilitation. BMC Cancer. 2019;19(1):1-11.

29. Van Rooijen SJ, Molenaar CJL, Schep G, Van Lieshout RHMA, Beijer S, Dubbers R, et al. Making Patients Fit for Surgery: Introducing a Four Pillar Multimodal Prehabilitation Program in Colorectal Cancer. Am J Phys Med Rehabil. 2019;98(10):888-96.
