# Supplementary material for: Effects of Prehabilitation Concurrent Exercise on Functional Capacity in Colorectal Cancer Patients: A Systematic Review and Meta-Analysis
Source: Healthcare (Basel). 2025 May 12;13(10):1119. doi: 10.3390/healthcare13101119 (PMC12110785; doi:10.3390/healthcare13101119)
Supplement: Supplementary file 1 [file healthcare-13-01119-s001.zip › Supplementary file 2.pdf]

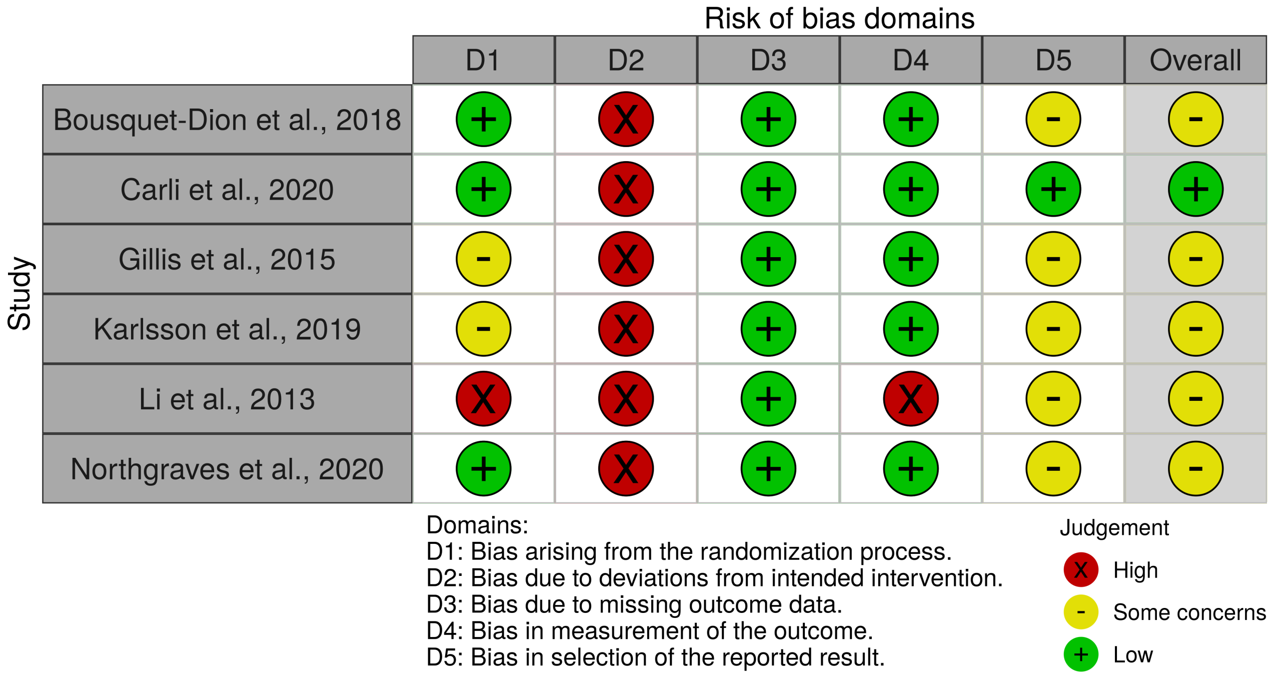
Risk of bias domains
D1
D2
D3
D4
D5
Overall
Study
Bousquet-Dion et al., 2018
Carli et al., 2020
Gillis et al., 2015
Karlsson et al., 2019
Li et al., 2013
Northgraves et al., 2020
Domains:
D1: Bias arising from the randomization process.
D2: Bias due to deviations from intended intervention.
D3: Bias due to missing outcome data.
D4: Bias in measurement of the outcome.
D5: Bias in selection of the reported result.
Judgement
High
Some concerns
Low

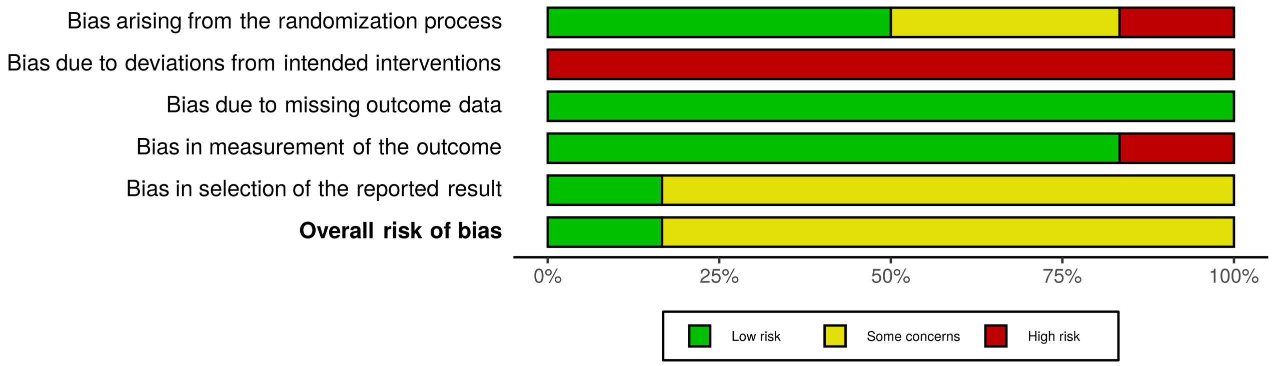
Bias arising from the randomization process
Bias due to deviations from intended interventions
Bias due to missing outcome data
Bias in measurement of the outcome
Bias in selection of the reported result
Overall risk of bias
0%
25%
50%
75%
100%
Low risk
Some concerns
High risk
