# Supplementary material for: Effects of Prehabilitation Concurrent Exercise on Functional Capacity in Colorectal Cancer Patients: A Systematic Review and Meta-Analysis
Source: Healthcare (Basel). 2025 May 12;13(10):1119. doi: 10.3390/healthcare13101119 (PMC12110785; doi:10.3390/healthcare13101119)
Supplement: Supplementary file 1 [file healthcare-13-01119-s001.zip › Supplementary file 3.pdf]

Supplementary file S2. Summary of meta-analysis findings and GRADE quality evidence synthesis.

outcome	Summary of findings				Quality of evidence synthesis (GRADE)				
	k	n	Effect (95% CI)	Direction effect compared to Control	Imprecision	Inconsistency	Risk of bias	Indirectness	Overall quality
					**Distance (Meters)**				
6 MWT	6	351	0.28 [0.03 to 0.88]	↑	-1	-1	-1	None	●●○○○ Low
