# Supplementary material for: Effects of Prehabilitation Concurrent Exercise on Functional Capacity in Colorectal Cancer Patients: A Systematic Review and Meta-Analysis
Source: Healthcare (Basel). 2025 May 12;13(10):1119. doi: 10.3390/healthcare13101119 (PMC12110785; doi:10.3390/healthcare13101119)
Supplement: Supplementary file 1 [file healthcare-13-01119-s001.zip › Supplementary File 6.pdf]

Supplementary File S5. Results for right-skewness test and flatness test for 6MWT distance.

	pBinomial	zFull	pFull	zHalf	pHalf
Right-skewness test	0.125	-3.862	0.00	-3.365	0.000
Flatness test	1.000	2.331	0.99	3.007	0.999

Note: p-values (pHalf) of 0 or 1 correspond to $p<0.001$ and $p>0.999$, respectively. Power estimate = 91% (55.8%, 99%). Evidential value present: yes. Evidential value absent/inadequate: no
