# Supplementary figures and images for: Effects of Prehabilitation Concurrent Exercise on Functional Capacity in Colorectal Cancer Patients: A Systematic Review and Meta-Analysis
Source: Healthcare (Basel). 2025 May 12;13(10):1119. doi: 10.3390/healthcare13101119 (PMC12110785; doi:10.3390/healthcare13101119)

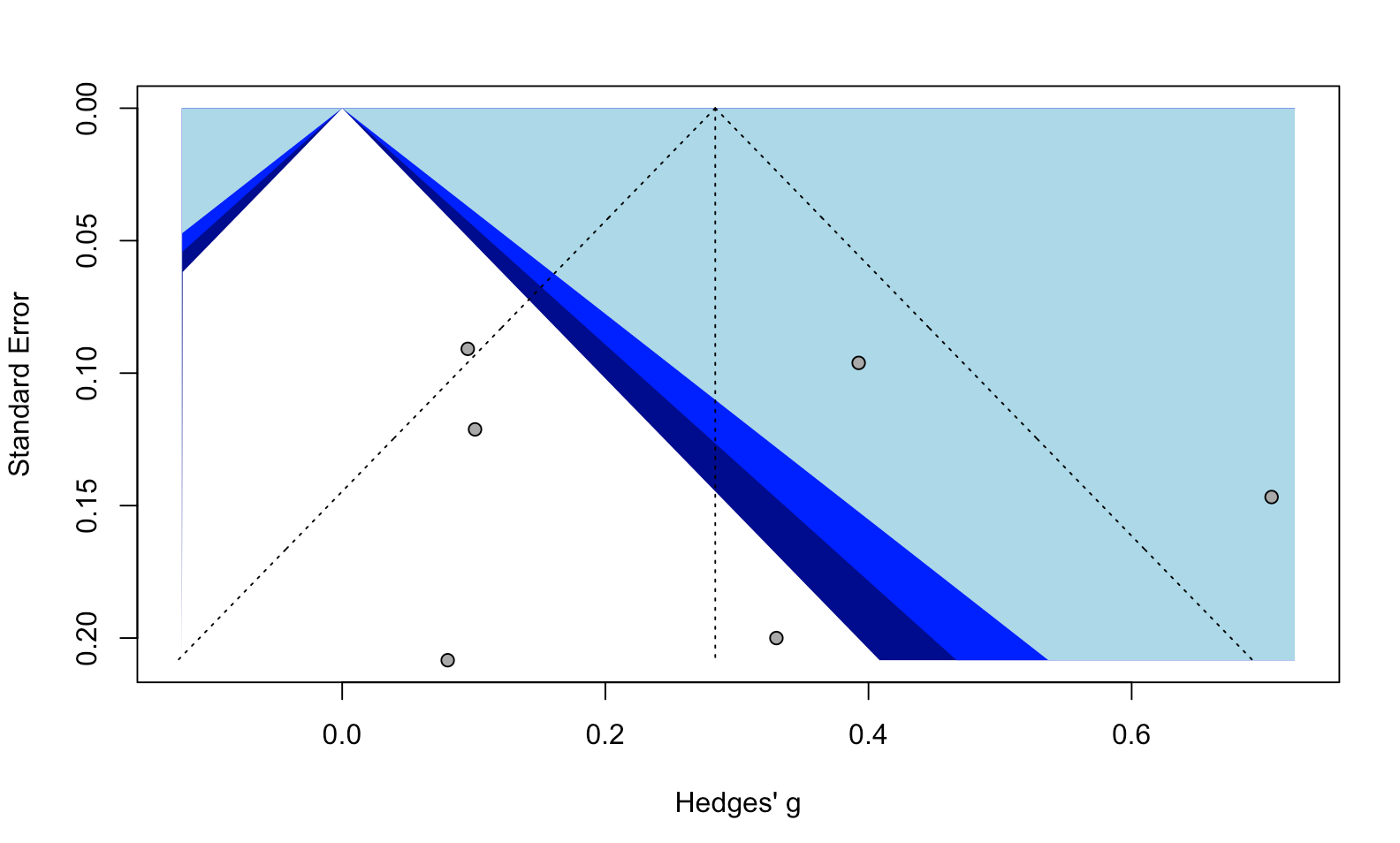

Supplement: Supplementary file 1 [file healthcare-13-01119-s001.zip › Supplementary file 4.png]

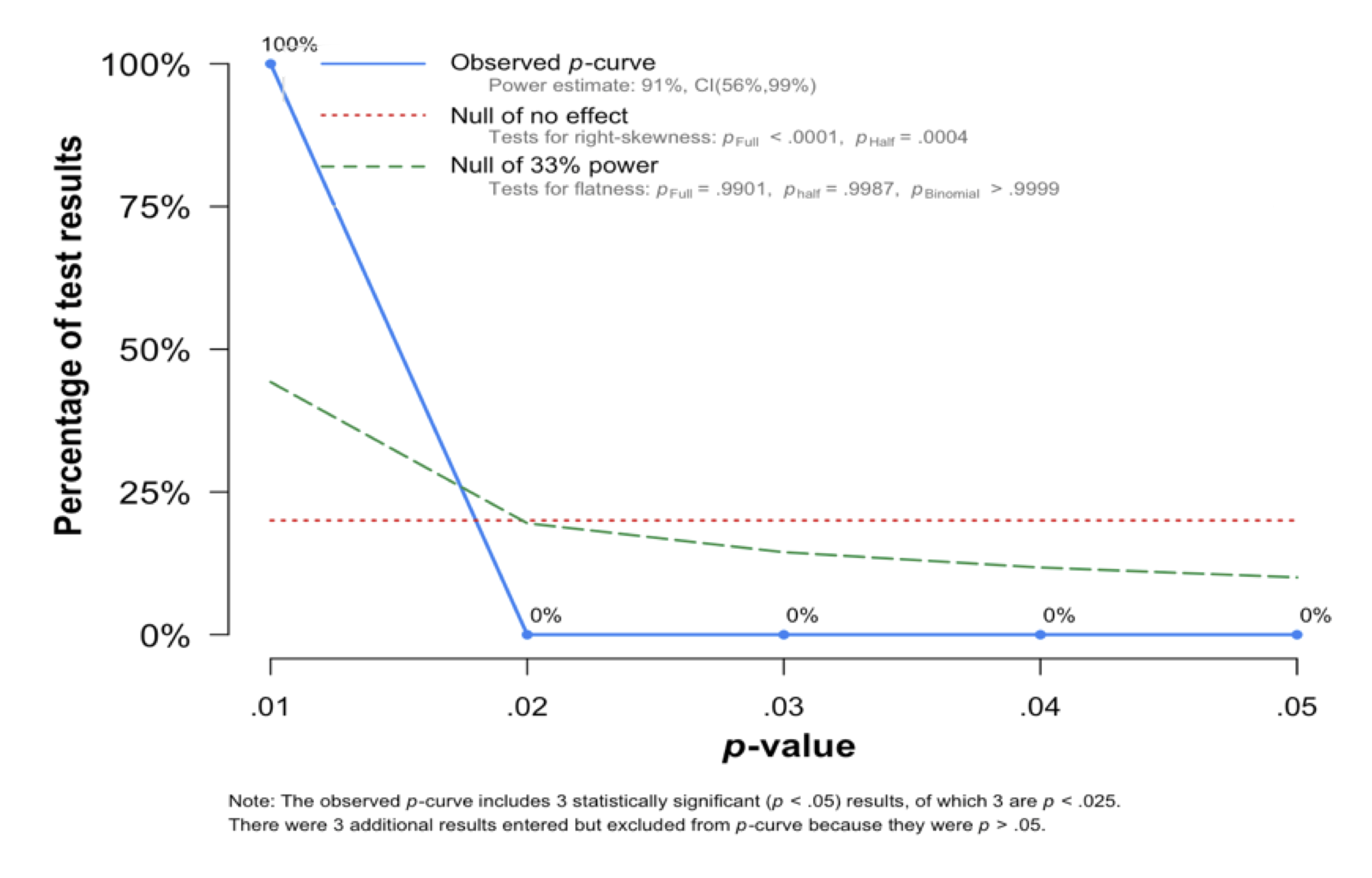

Supplement: Supplementary file 1 [file healthcare-13-01119-s001.zip › Supplementary file 5.png]
